# Challenges to estimating and managing risks with hexavalent chromium exposure: a mixed-methods study of Swedish workplaces

**DOI:** 10.1093/annweh/wxaf039

**Published:** 2025-07-04

**Authors:** Linda Schenk, Malin Engfeldt, Håkan Tinnerberg, Niklas Ricklund, Martin Tondel, Pernilla Wiebert, Maria Albin, Karin Broberg

**Affiliations:** Institute of Environmental Medicine, Karolinska Institutet, Stockholm, Sweden; Division of Occupational and Environmental Medicine, Department of Laboratory Medicine, Lund University, Lund, Sweden; Department of Occupational and Environmental Medicine, Region Skåne, Lund, Sweden; Occupational and Environmental Medicine, Sahlgrenska University Hospital, Gothenburg, Sweden; Occupational and Environmental Medicine, School of Public Health and Community Medicine, Institute of Medicine, University of Gothenburg, Gothenburg, Sweden; Department of Occupational and Environmental Medicine, Faculty of Medicine and Health, Örebro University, Örebro, Sweden; Department of Occupational and Environmental Medicine, Uppsala University Hospital, Uppsala, Sweden; Occupational and Environmental Medicine, Department of Medical Sciences, Uppsala University, Uppsala, Sweden; Institute of Environmental Medicine, Karolinska Institutet, Stockholm, Sweden; Centre for Occupational and Environmental Medicine, Region Stockholm, Stockholm, Sweden; Institute of Environmental Medicine, Karolinska Institutet, Stockholm, Sweden; Centre for Occupational and Environmental Medicine, Region Stockholm, Stockholm, Sweden; Division of Occupational and Environmental Medicine, Department of Laboratory Medicine, Lund University, Lund, Sweden

**Keywords:** bath plating, carcinogens, mutagens and reprotoxic substances directive, occupational exposure limit values, steel production, welding

## Abstract

Using a mixed-methods approach, we assessed understanding of risks from exposure to the non-threshold carcinogen hexavalent chromium (Cr(VI)) among workers (*n* = 113) and occupational health and safety managers (*n* = 13) at 14 worksites with potential exposure to Cr(VI). We found that 55% of the workers had a measurable concentration of inhalable Cr(VI), with 19% exceeding 1 µg/m^3^, a level that corresponds to an “upper risk level” for future EU binding occupational exposure limits over a working lifetime. Additionally, 52% of workers had red blood cell (RBC) Cr concentrations exceeding the 95th percentile of an unexposed control group. Among responding workers (*n* = 91), 35% reported to perceive to be at no or low risk due to Cr(VI) exposure, 47% to be at some or large risk while 18% stated to be unsure. No correlations were found between reported risk perceptions and measured inhalable Cr(VI), urinary Cr, or RBC-Cr, but a weak correlation to years employed was found. Observations indicated that the hierarchy of controls was not strictly followed. Furthermore, 42% of respiratory protective equipment users used it incorrectly, and only two out the 50 (4%) needing a fit-test reported having performed one. Interviews with the managers revealed a lack of knowledge about the health risks of Cr(VI), and that expectations about exposure levels did not always match measured exposures. Our findings identify knowledge gaps regarding the health hazards of Cr(VI) and highlight the difficulty of estimating workplace exposure and risk without measurements. Based on our findings we recommend efforts to improve knowledge about Cr(VI) health hazards, strengthen the adherence to the hierarchy of controls, and incentivize quantitative exposure assessments.

**What’s important about this paper?**

Exposures to hexavalent chromium in the range of a single microgram per cubic meter of air are associated with a significant risk of cancer, so there is a need for a risk management strategy that reduces exposures to *as low as reasonably practicable*. This study investigated risk management practices, understanding, and knowledge needs in Swedish workplaces with hexavalent chromium and found clear knowledge gaps about health hazards and gaps between perceived and actual exposure risks.

## Introduction

Occupational exposure to hexavalent chromium, Cr(VI), occurs in the production, processing, and casting of stainless steel and other iron-chrome alloys, welding in stainless steel, production, and use of chromates (eg anti-corrosives, pigments, and wood-impregnation), chrome-plating and other metal surface treatments (SCOEL 2017). Cr(VI) is a human carcinogen, causing an increased risk of lung cancer and possibly other cancers (den Braver-Sewradj et al. 2021). Other potential adverse health effects include, for example, kidney toxicity, allergic contact dermatitis, respiratory symptoms, skin corrosion, asthma, depending on concentration, duration of exposure, and exposure routes (Hessel et al. 2021). Further, we have identified lung-cancer-related molecular changes (Jiang et al. 2024b, 2025) even at exposure to Cr(VI) levels below the current OEL. The carcinogenicity of Cr(VI) is assumed to act through non-threshold mechanism(s) (SCOEL 2017; den Braver-Sewradj et al. 2021), not allowing the determination of a strictly health-based occupational exposure limit (OEL). It has been estimated that 40 years of occupational inhalation exposure to 5 μg/m^3^, the Swedish OEL at the time of study, would yield 20 extra lung cancer cases per 1000 exposed workers (SCOEL 2017). A lowering of the Swedish OEL to 1 μg/m^3^ has recently been proposed. It can be noted that the European Commission’s Advisory Committee on Safety and Health at Work (2022) adopted the policy that future EU OELs for non-threshold substances will be set between an “upper risk level” and “lower risk level.” The upper risk level is 4 predicted cancer cases in 1,000 employees and the lower risk level is 4 in 100,000, assuming exposure occurs over 8 hours per day, 5 days a week, and 40 years of working life. Drawing on SCOEL (2017), the exposure range between the lower and upper risk levels for Cr(VI) would be 0.01 µg/m^3^ to 1 µg/m^3^. For certain occupations, notable fractions of the European workforce may exceed the upper-risk level (Santonen et al. 2022; Jiang et al. 2024a), including trainees at vocational schools (Saber et al. 2025).

In Sweden, the number of workers exposed to Cr(VI) has remained relatively constant over several decades. (Kauppinen et al. 2002) estimated 21,000 Swedish workers in the 1990s and Jiang et al. (2024a) estimated 17,900 today. Given the low air concentrations that correspond to the upper-risk level and that the number of exposed workers is not decreasing, studies of risk management practice and how to improve mitigation of Cr(VI) risks are warranted.

The Swedish provisions on Risks in the Work Environment (AFS 2023a), contain the transposed rules from the Chemical Agents Directive (EC 1998) and the Carcinogens, Mutagens and Reproductive Toxicants Directive (EC 2004). The provisions outline a hierarchy of controls, where (i) elimination and (ii) substitution are primarily preferred measures. The provisions also list, in order of priority and efficiency: (iii) enclosure or encapsulation of the substance or work process, (iv) use work methods, processes, or tools that limit exposures as far as possible, (v) process ventilation or other technical measures at the source, (vi) minimize the number of persons exposed, and (vii) use of personal protective equipment (PPE). PPE is a last resort if other measures are insufficient to control risks. For chemical products that fulfil classification criteria for carcinogens as well as for certain work processes, elimination, substitution, and enclosure or encapsulation are required steps if technically possible.

Research indicates that individuals’ perception of hazards and their own vulnerability affect which precautions they take. Within the area of occupational safety, a systematic review by Priolo et al (2025) noted that a majority of studies found a positive relationship between risk perception and safety behaviour. For instance, workers’ risk perception regarding noise predicted their use of hearing protection, although they did not correctly assess their exposure and level of risk (Arezes and Miguel 2008). Additionally, research on chemical exposure shows that workers do not know their exposure levels (Pui et al. 2017) and that both experts’ and workers’ self-assessed exposures are inaccurate (Vadali et al. 2012; Arnold et al. 2016; Coşkun Beyan et al. 2023). That being the case, without measurements, workers and managers may lack adequate estimates of their workplace exposures and associated risks.

Moreover, factors other than exposure to a risk source impact personal risk perception. Amongst others, gender influences risk perception; women tend to perceive the same risks as higher than men (Gustafson 1998). Expanding on this topic, other studies have identified a “white male effect,” i.e. that a society’s privileged groups perceive risks as lower (Finucane et al. 2000). Another observed phenomenon is unrealistic optimism, referring to people generally perceiving risks to themselves as lower than to others (Shepperd et al. 2015). Delay-discounting, meaning that health effects with a long delay between exposure and occurrence are discounted compared to more immediate effects or benefits, can also influence risk perception (Odum et al. 2020) . One might thus expect occupational risks of Cr(VI) to be underestimated.

Nevertheless, understanding risks and how to effectively mitigate them is a prerequisite for workers and managers to make informed decisions, especially for exposures connected to serious health effects with a long latency, such as carcinogenicity. However, it can be challenging to measure understanding of such risks. Weinstein (1999) concluded “that decisions about personal risks require, at a minimum, information about the nature and likelihood of potential ill effects, information about risk factors that modify one’s susceptibility, and information about the ease or difficulty of avoiding harm.” In the case of Cr(VI) exposure, this corresponds to an understanding of the health hazards of Cr(VI), dose-response for those hazards, an understanding of personal exposure, and how exposure can be reduced.

The primary aim of the present study was to investigate the understanding, perception, or assessment of risks with exposure to Cr(VI). This was also set in relation to measured exposures and current requirements according to the Occupational Safety and Health (OSH) legislation. Additionally, we also aimed to map out potential knowledge needs. To achieve these aims, we employed a convergent parallel mixed-methods design, which included questionnaires and exposure assessments for workers as well as interviews with their OSH managers.

## Methods

This study was part of a cross-sectional study of Cr(VI) exposure in the Swedish work environment, carried out by all the seven Occupational and Environmental Medicine clinics in Sweden (Lund, Gothenburg, Linköping, Örebro, Stockholm, Uppsala, and Umeå) and associated university divisions of Occupational and Environmental Medicine. Four streams of data are analysed:

- personal exposure measurements of workers (also reported in Jiang et al. 2024a),
- observations about exposure mitigation measures at workplace visits,
- questionnaires distributed to workers on risk assessment and risk mitigation measures,
- interviews with representatives of workplace risk management.

Companies and study participants were recruited from June 2021 through May 2022 (see also Jiang et al. 2024a), by occupational hygienists at each clinic. Companies were recruited based on the potential for Cr(VI) exposure and participants had to be non-smokers (>6 months). 14 worksites from 13 companies participated in the study, the number of employees in production at each worksite ranged from below ten to over one-thousand. In addition, 72 workers who were not current smokers and not occupational exposed to Cr(VI) or other genotoxic substances were recruited as a control group (Jiang et al. 2024a).

Participants’ exposure to Cr(VI) was assessed by personal air sampling of inhalable Cr(VI), outside respiratory protective equipment (RPE), during a workday and biomonitoring of chromium (Cr) in red blood cells (RBC-Cr) and in pre- and post-shift urine samples during the same day (urinary Cr). The sampling was performed after at least three working days. RBC-Cr represents Cr(VI) whereas urinary Cr represents both Cr(III) and Cr(VI). Furthermore, RBC-Cr represents exposures during approximately the past four months, while urinary Cr mostly represents exposure during the past days (Ndaw et al. 2022 ; Santonen et al. 2022). Urinary Cr values presented herein are the density-adjusted Cr concentrations, calculated as follows: C_adjusted_ = C_measured_ × (1- ρ_mean_)/(1-ρ_sample_), where C_measured_ = Cr concentration in the sample, ρ_mean_ = average urinary density of all participants, and ρ_sample_ = density of the urine sample. The limit of detection (LOD) for inhalable Cr(VI) was 0.08 µg per sample (for our sampling duration and pump settings this corresponds to air concentrations below 0.04 µg/m^3^) and 0.20 μg Cr /L in both blood and urine. Details on sampling and chemical analysis, as well as creatine-adjusted Cr concentration in urine, are reported in Jiang et al. (2024a).

For our analyses, the results of the exposure measurements were grouped into low, medium, and high exposure. For inhalation, the ACSH (2022) policy on “low risk” and “high risk” was combined with the exposure: risk relationship derived by SCOEL (2017) to identify 1µg/m^3^ as corresponding to high risk and 0.01 µg/m^3^ to low risk, assuming a work-life’s exposure. Hence exposures above 1 µg/m^3^ were categorized as high. As 0.01 µg/m^3^ would yield a Cr(VI) amount below the LOD, below LOD is the closest proxy for corresponding to an acceptably low risk and was selected as the cut-off for categorizing inhalation exposures as low. There are no established exposure:risk relationships for urinary Cr or RBC-Cr. Hence, we used the 95th percentiles (P95) of the unexposed control group in Jiang et al. (2024a) as the upper limit for low exposures: 0.53 µg Cr/L in post-shift urine (density adjusted) and 0.72 µg/L RBC-Cr. As a cut-off for high exposure, we selected the P85 of the exposed participants: 2.13 µg Cr/L in post-shift urine (density adjusted) and 1.18 µg/L RBC-Cr.

During the sampling day, occupational hygienists documented exposure mitigation measures and judged their effectiveness based on visual observations. The occupational hygienists also collected information about the types of RPE participants used, such as Powered Air Purifying Respirator (PAPR) or tight-fitting types. Observations were reported in a standardized form developed by partners from all clinics for this study. Sufficient engineering controls were defined as general ventilation, process ventilation, or enclosure efficient enough to warrant no use of RPE (regardless of whether RPE was used or not). Correct use of RPE was defined as: Reporting to use RPE when needed, using the correct type of filter, reporting regular filter changes, and correct storage. The worksites were grouped into the categories of bath plating, manufacture/processing of metal products, steel production, and other applications. The main work task of the participants was grouped into welding, process operation, machining, and others (see Jiang et al. 2024a).

Each participant was also asked to fill in a questionnaire containing questions on demographics, work tasks, preventive measures, hazards, and perceived risk (see also Supplementary Material S1). The presence or absence of specific preventive measures were asked as Yes/No questions. The questionnaire item on hazards contained six health outcomes. Four of which are established hazards of Cr(VI): cancer in the lung and airways, cancers in other parts of the body, eczema, and other lung diseases. Two diseases, i.e. cardiovascular disease and osteoporosis, not associated with Cr(VI) exposure according to the current state of knowledge, served as negative controls. The items on participants’ perception or opinion on preventive measures or risk were on a five-level scale, and the item on perceived risk additionally had an “unsure” option. The questionnaire was sent to participants in advance and collected during the biological sampling, when they were also given the opportunity to ask for any clarifications needed.

The filled-in questionnaires and coding sheets were transferred to Excel for cross-tabulations, while statistical analyses were performed in R (ver 4.4.0, R Core Team 2024). Stuart’s τ_c_ was calculated using the DescTools package (Signorell 2025). Relative risks were calculated using a modified Poisson regression approach with bootstrapped confidence intervals (1000 iterations), using the rqlm package (Noma 2025).

Each participating company was also asked to identify a representative for OSH issues, to interview about the workplace risk management of Cr(VI) exposures. Interviews were conducted prior to the sampling day, with a follow-up interview after the air measurements had been reported back to the company. Interview guides are provided in Supplementary Material S2. The first interviews took 40 to 60 min and the second 10 to 30 min. Interviews were recorded and summarized in text format according to the major themes of the interview guide. All interviews and summaries were performed by one person (LS). Interviewees were asked to read and provide potential corrections or clarifications to the summaries. Structural coding was performed on the interview summaries through repeated close readings (Saldana 2021). For the present study, we extracted the material pertaining to risks due to Cr(VI) at the workplace, risk mitigation measures, and the hierarchy of controls, as well as information needs. The extracted material was analysed thematically with a low level of abstraction (Saldana 2021), following three deductive themes: perception of Cr(VI) risk, views on the hierarchy of controls, and expressed information needs. An analysis of the remaining material will be presented elsewhere.

Informed consent was collected from all study participants. The study was approved by the Swedish Ethical Review Authority (Dnr 2021-00641).

## Results

In total, 12 out of 14 companies participated in both the measurement and questionnaire parts as well as the two interview parts of the study. One company did not participate in the second interview (company 14) and one company declined participation in both interview parts (company 1). An overview of workplace categories, interviewees’ function, and number of participating workers are given in Table 1.

**Table 1.** Overview of type of industries, interviewee functions, number of workplace measurements, and study participants by work-task

Sector and company number	Interviewee function	Number of participating workers	Participants per work-task			
			Machining	Process operator	Welding	Other
Bath plating						
1	Declined	9	-	5	-	4
2	EHSQ manager	5	3	2	-	-
3	Production manager	3	2	1	-	-
Manufacture/processing of metal products						
4	Production manager	6	-	-	6	-
5	EHSQ staff	11	2	1	7	1
6	EHSQ staff	8	-	1	7	-
7	EHSQ manager	10	3	2	3	2
8	EHSQ staff	6	-	-	3	3
9	EHSQ staff	6	-	6	-	-
10	EHSQ staff	9	1	4	4	-
Steel production						
10	*Same person as above* ^a^	15	-	15	-	-
11	EHSQ manager	10	-	6	-	4
12	EHSQ staff	7	-	7	-	-
Other applications						
13	Production manager^b^	6	-	5	-	1
14	EHSQ staff^c^	2	-	2	-	-

EHSQ—Environment, Health, Safety and Quality (N.B. some participants used parts of the label in their title, e.g. EHS or synonym titles).

^a^Company 10’s operations covered two worksites belonging to different workplace categories, participating workers have been divided according to category, one person from the EHSQ section was interviewed for worksites.

^b^Two EHSQ staff members participated in the interview.

^c^Declined the second interview.

From each worksite, 2 to 24 workers participated in the exposure assessment with inhalable dust, urinary Cr, and RBC-Cr. The largest share of participants were process operators (*n* = 57), followed by welders (*n* = 30). The majority of companies directed the interview request to the companies’ function for environment, health, safety, and quality (EHSQ), and in a few cases to the production manager of the site. Interviewees’ ages ranged from 28 years to 60 years (data not shown), and participants responding to the questionnaire and participating in the exposure assessment ranged from 20 years to 67 years old (Table 2). None of the exposed workers reported to ever have been diagnosed with cancer (data not shown).

**Table 2.** Overview of participants, workplace observations, questionnaire responses, and exposure markers for hexavalent chromium (Cr(VI)). We report number of positive answers over number of responses for each question as well as percentage, due to baseline population differences and missing data.

	Women (*n* = 15)	Men (*n* = 98)	Total (*n* = 113)
Median age (range):	40 (20–56)	38 (20–67)	39 (20–67)
Exposure measures^f^ geometric mean (geometric standard deviation)			
Inhalable Cr(VI) µg/m^3^	0.11 (7.2)	0.16 (6.9)	0.16 (6.9)
Urinary Cr ^density adjusted^ pre-shift µg/L	0.36 (2.7)	0.51 (3.1)	0.49 (3.0)
Urinary Cr ^density adjusted^ post-shift µg/L	0.49 (5.3)	0.67 (2.9)	0.64 (3.2)
Red blood cell Cr µg/L	0.66 (1.3)	0.83 (1.5)	0.81 (1.5)
Risk mitigation, observed (^obs^) and reported			
Engineering controls sufficient^obs^ ^a^	13/15 (87%)	60/98 (61%)	73/113 (65%)
…of those >LOD_inhal_^b^	6/7 (86%)	28/55 (51%)	34/62 (55%)
Used any RPE^obs^	10/15 (67%)	41/98 (42%)	51/113 (45%)
…of those >LOD_inhal_^b^	7/7 (100%)	30/55 (55%)	37/62 (60%)
Used their RPE correctly^obs^	8/10 (80%)	22/41 (54%)	30/51 (59%)
…of those >LOD_inhal_^b^	6/7 (85%)	19/55 (35%)	25/62 (40%)
Report to never use any RPE^obs^	3/15 (20%)	14/98 (14%)	17/113 (15%)
… of those >LOD_inhal_^b^	0/7 (0%)	8/55 (15%)	8/62 (13%)
Only uses PAPR as RPE^obs^	8/15 (53%)	38/98 (39%)	46/113 (41%)
Performed fit test RPE^d^	1/4 (25%)	1/46 (2%)	2/50 (4%)
Received instruction	12/15 (80%)	64/93 (69%)	76/108 (70%)
Perception of risk mitigation, reported			
Considers own knowledge of working safely sufficient	9/12 (75%)	49/79 (62%)	58/91 (64%)
Considers that conditions for safe work are provided	10/12 (83%)	47/79 (59%)	57/91 (63%)
Perceives to be at risk (some-very large) due to Cr(VI)	7/12 (58%)	31/79 (39%)	38/91 (42%)
Knowledge of health risks^e^, reported			
Cancer in lungs and airways	12/12 (100%)	69/76 (91%)	81/88 (92%)
Cancer in other parts of body	8/12 (67%)	40/76 (53%)	48/88 (55%)
Eczema	4/12 (33%)	22/76 (29%)	26/88 (30%)
Other lung diseases	3/12 (25%)	42/76 (55%)	45/88 (51%)
Osteoporosis^‡^	0/12 (0%)	5/76 (7%)	5/88 (6%)
Cardiovascular disease^‡^	0/12 (0%)	5/76 (7%)	5/88 (6%)

LOD—Limit of detection; LOD_inhal—_LOD for inhalable Cr(VI); PAPR—Powered Air Purifying Respirator (a subset of RPE); RPE—Respiratory Protective Equipment.

^obs^– based on occupational hygienists’ observations during day of exposure assessment.

^‡^False statements used as a negative control since these outcomes have not been linked to Cr(VI).

^a^A combined assessment of enclosure/encapsulation and process ventilation.

^b^7 women and 55 men had inhalable Cr(VI) levels above LOD, measurements performed outside of RPE.

^c^Note that reporting to only use PAPR does not mean always using.

^d^According to occupational hygienists’ data collection during measurement days, 50 participants (4 women and 46 men) sometimes use tightfitting RPE, thus needing a fit-test.

^e^It should be noted that two ticked yes for all six health risks (both men).

^f^Samples under LOD (45% for inhalable (Cr(VI), 21% for pre-shift and 17% for post-shift Urinary Cr and 0% for RBC-CR) were replaced by LOD/2 for calculations of inhalable Cr(VI) concentrations, density adjusted urinary Cr, geometric means and minimal exposure values.

First, we present the results from exposure measurements, observations, and questionnaires. In the final three sections, we present the data from interviews. Unfortunately, responses regarding knowledge of hazards and perceived risks were missing from a substantial share of participants. Hence, the number of responses is given separately in tables and figures.

### Exposure measurements, observed risk mitigation, and questionnaire results

The average geometric means of exposures were 0.16 µg/m^3^for inhalable Cr(VI), 0.49 µg Cr/L in pre-shift urine (density adjusted), 0.64 µg Cr/L in post-shift urine (density adjusted) and 0.81 µg Cr/L in RBC (Table 2). Variability was the highest for inhalable Cr(VI) and smallest for RBC-Cr (Table 2). For all exposure metrics the category of ‘low exposure’ was the most frequent (see Supplementary Table S3.1). For inhalable Cr(VI) 45% of participants (*n* = 51) were grouped in the low exposure group (ie below LOD), and 19% (*n* = 22) in the high-exposure group, above 1 µg/m^3^. For urinary Cr, 53 participants (47%) were below our cut-off for the low-exposure group, the P95 of the unexposed control group, and 17% of post-shift urinary Cr were below the LOD. For RBC-Cr, 59 out of 113 (52%) were categorized into the low exposure group (0.72 µg/L), no samples were below the LOD for RBC-Cr (Supplementary Table S3.1).

Although the number of women was low (*n* = 13), this small group displayed a slightly different pattern, reporting more frequent and correct use of RPE than men (Table 2). The observations showed that far from all participants’ workplaces were judged by the occupational hygienists to have sufficient engineering controls (65%) or used any type of RPE (45%). Even fewer were assessed to use the RPE correctly (58% of RPE users), and only 2 of the 50 participants sometimes using tight-fitting RPE reported having performed a fit-test for their RPE. Occupational hygienists to a higher degree categorized engineering controls as sufficient for participants in the low exposure group for inhalable Cr(VI) (76% compared to 55% in the medium and high exposure groups). Nevertheless, among the 73 participants categorized as having sufficient engineering controls, 34 (46%) did have inhalable Cr(VI) exposure above the LOD and 14 of these (19%) were in the high exposure group exceeding 1 µg/m^3^.

A majority of participants reported having received instruction about safe working practices, however, it is noteworthy that 30% reported not having received such instruction. Similar numbers were seen for the questions about how participants perceived the sufficiency of their own knowledge and conditions provided by the workplace. No worksite had 100% insufficiently informed participants (data not shown). Forty-two percent perceived themselves to be at some or large risk due to Cr(VI) exposure. The share reporting that they judged to be at some or large risk for health effects due to Cr(VI) exposure ranged from 0% to 88% across workplaces. The lung-cancer risk of Cr(VI) was well known, while the potential for cancer in other organs, eczema, and other lung diseases was less so. Very few identified the negative controls, that is the two diseases not associated with Cr(VI) (Table 2). Eight out of 88 (9%) correctly identified the four health effects connected to Cr(VI), and none of the negative control effects (data not shown).

**Fig. 1.**
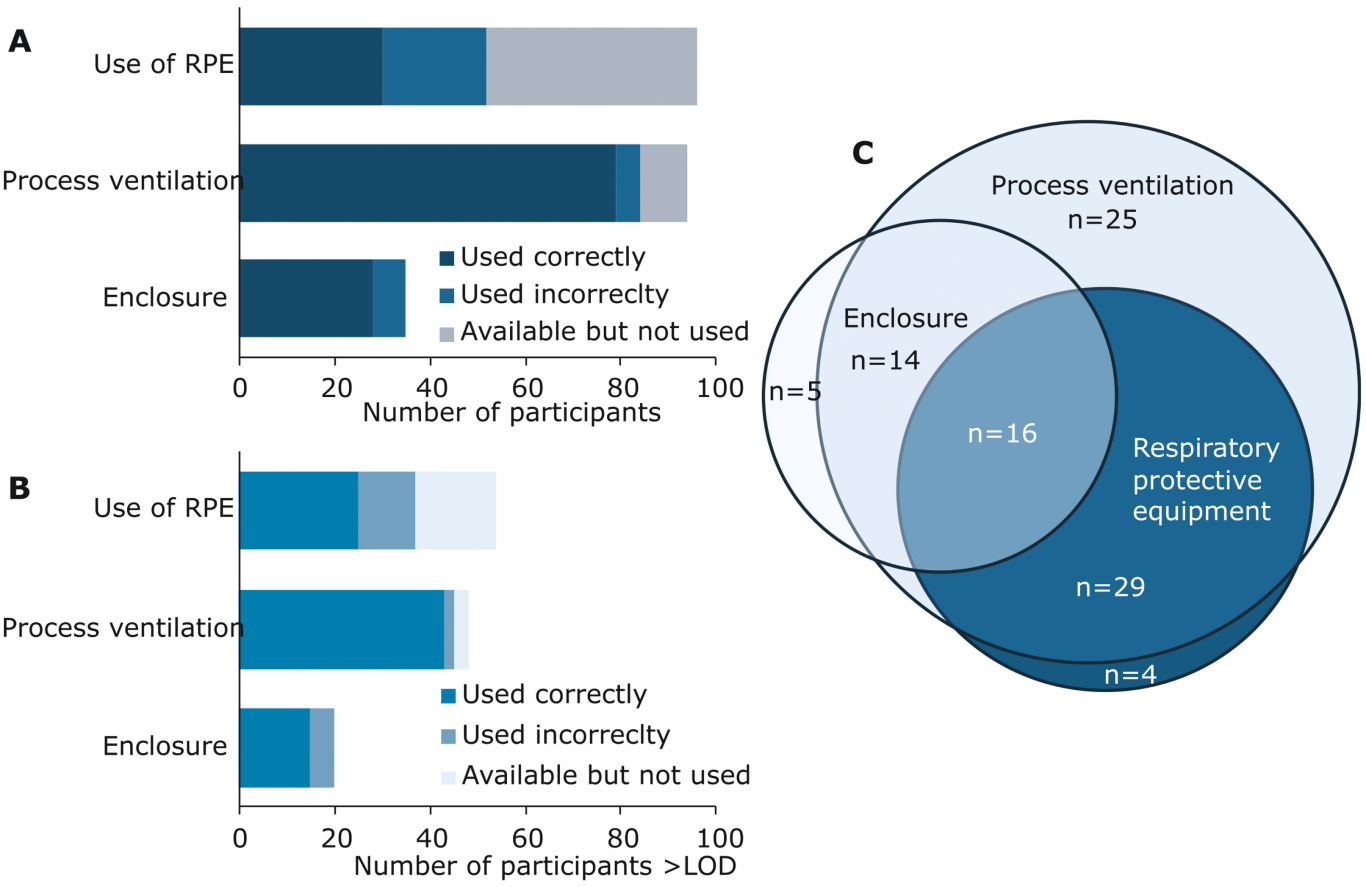
Overall use of exposure reduction measures by individual participants during measurement days according to occupational hygienists’ observations. Panel A displays all participants (*n* = 113), measures are listed from the bottom in order of hierarchy of controls specified in the provisions of chemical hazards, with the least preferred measure on top. Each participant could employ more than one measure. Panel B displays the same information but only for participants whose inhalable Cr(VI) levels exceeded the LOD (*n* = 62). Panel C displays the overlap in used exposure reduction measures for all participants using at least one exposure mitigation measure during the measurement days (*n* = 93). LOD—limit of detection; RPE—Respiratory Protective Equipment (any type).

Process ventilation, in the form of local exhaust ventilation (LEV) was the most frequently employed exposure reduction measure, followed by the use of RPE. The enclosure was the least used of the three measures. The pattern was the same for only participants with inhalable Cr(VI) above LOD. Minimizing the number of exposed workers (third in priority) was not assessed during the workplace visits. Most participants used at least one of the investigated exposure reduction measures (82%). Of these four participants (4%) only used RPE, and 16 (14%) used all three levels shown in Fig. 1.

**Fig. 2.**
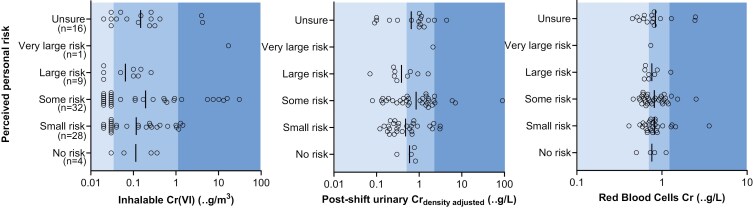
Perceived risk plotted over measured exposures for each participant (n=91), note the logarithmic scales on the x-axes. Colour coding indicating from left to right: low (light blue), medium, and high (dark blue) risk, see methods. Samples under LOD (45% for inhalable (Cr(VI) and 17% for post-shift Urinary Cr) were replaced by LOD/2 for calculations of inhalable Cr(VI) concentrations and density-adjusted urinary Cr.

Figure 2 shows the participant’s perceived risk for health effects due to Cr(VI) exposure plotted over the three exposure measures. A substantial share (18%) reported to be uncertain about their level of risk (top row in each panel, see also Supplementary Fig. S3.1). There was no clear correlation between exposure measures and reported risk perception (inhalable Cr(V) τc = −0.032, 95%CI −0.194 to 1.129; post-shift urinary Cr τc= 0.092, 95%CI +-0-075-0.259; RBC-CR τc = −0.035, 95%CI −0.196 to 0.126, see also Supplementary Table S3.2).

There were differences depending on workplace category and type of work tasks (Table 3). Employees from bath plating companies were less likely than respondents from other companies to report to be at some or large risk due to Cr(VI) exposure. For work tasks, machining was most frequent within bath plating, and less likely to report to be at some or large risk, while welders were slightly more likely to report being at risk. No difference was found regarding the observed sufficiency of engineering controls. Participants reporting to, at least sometimes, use tight-fitting RPE, were less likely to state to be at some or large risk than those stating to never use RPE or exclusively use PAPR. No differences based on perceived sufficiency of knowledge about safe working practices were seen, while participants stating to not be provided with the conditions for safe work were somewhat more likely to report to be at some or large risk due to Cr(VI) exposure (Table 3). No trends were seen regarding age and perceived risk (Supplementary Fig. S3.2), but median number of worked years was slightly higher for participants reporting to be at large or very large risk (τ_c_ = 0.262, 95%CI 0.0894 to 0.434, excluding unsure participants, Supplementary Fig. S3.3).

**Table 3.** Cross-tabulations between perceived risk due to Cr(VI) exposure and observed or self-reported workplace characteristics. For workplace characteristics, relative risk (RR) and 95% confidence interval (CI) of perceiving to be at some or large risk is calculated using the other 1-3 characteristics combined as reference, excluding participants reporting to be unsure.

	Perceived risk due to Cr(VI)			Row total	RR (95%CI) for percieved risk to be “Some-large”^b^	
	No-small	Some-large	Unsure			
*Sector*						
Bathplating	11	1	5	17	0.1	(0.02–0.5)
Manufacture/processing of metal products	16	33	6	55	1.8	(1.1–3.1)
Steel production	2	6	5	13	1.4	(0.7–5.3)
Other applications	3	3	0	6	-	-
*Work task*						
Machining	6	1	4	11	0.2	(0.03–0.9)
Process operator	13	15	7	35	0.9	(0.6–1.3)
Welding	5	22	3	30	1.9	(1.3–2.5)
Other	8	5	2	15	-	-
*Sufficiency of engineering controls* ^obs^ ^a^						
Sufficient	24	33	9	66	1.0	(0.7–1.8)
Insufficient	8	10	7	25	-	-
*Reported RPE use* ^obs^						
Never uses RPE	6	11	2	19	1.2	(0.7–1.8)
Only PAPR	12	24	5	41	1.4	(0.9–2.1)
Other types of RPE	14	8	9	31	0.6	(0.3–0.9)
*Own knowledge of working safely*						
Sufficient	24	25	8	57	0.7	(0.5.1.1)
Undecided	3	8	0	11	1.3	(0.8–2.8)
Insufficient	5	10	8	23	1.2	(0.7–1.8)
*Conditions for safe work provided*						
Yes	25	25	7	57	0.7	(0.5–1.0)
Undecided	6	6	4	16	0.9	(0.4–1.4)
No	1	12	5	18	1.8	(1.2–4.0)

PAPR—Powered Air Purifying Respirator; RPE—Respiratory Protective Equipment.

^obs^Based on occupational hygienists’ observations during measurement day.

^a^A combined assessment of encapsulation or enclosure, and process ventilation.

^b^Confidence intervals have not been adjusted for multiplicity. Model summaries are presented in Supplementary Table S3.2.

### Interviewees’ perception of Cr(VI) risks at the workplace

**Fig. 3.**
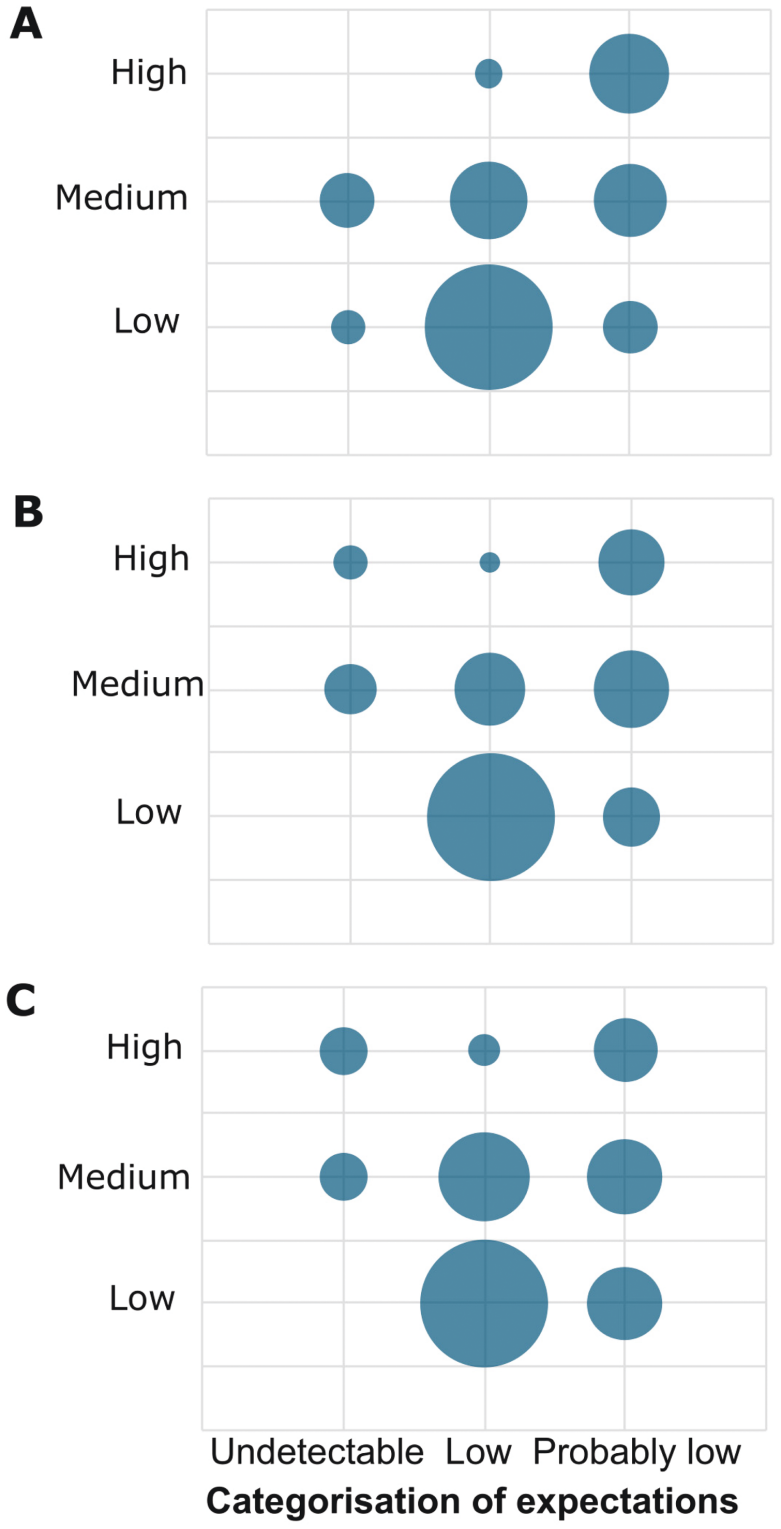
Interviewees pre-measurement expectations (x-axis) compared to participants' exposure group (y-axis), bubble size is proportional to the number of participants. **A)** Inhalation exposures, grouped according to whether exposures were above the estimated level for the upper-risk level (high) as defined in ACSH (2022), below the limit of detection (LOD; Low) or in between (Medium). **B**) Density-adjusted Cr in post-shift urine and **C)** Red blood cell Cr are both categorized as follows: Below the P95 of the unexposed control group in Jiang et al. (2024a) (Low), above the P85 of the exposed group (High) or in between (Medium). Note that an air concentration corresponding to the lower risk level would be below the LOD.

During initial interviews with workplace risk management representatives regarding Cr(VI) exposure risks, the focus was primarily on exposure levels, with the consensus that lower exposures correlate with lower risks. Based on the interviewees’ expectations of exposure levels prior to the measurements, workplaces were categorized into three groups: *Undetectable*, expecting exposures to be below the LOD. *Low*, expecting that exposures would be low in relation to current OEL. *Probably low*: noting recent efforts to improve ventilation, but expressing some uncertainty about whether exposure levels had been reduced to non-concerning levels. As shown in Fig. 3, there were substantial overlaps in measured exposures between the three groups.

Some interviewees also mentioned the absence of severe disease as a testament to the low risk for health effects, yet admitting this may be an insufficient line of argumentation. “We don’t have internal statistics of anyone actually becoming ill after 30 years in our [work-, *authors’ note*] environment. Although, we haven’t made any systematic investigations of lung function and capacity etcetera.” The health risks that were explicitly raised by one or several informants were cancer (unspecified), lung cancer, lung effects, cardiovascular disease, eczema (contact allergy), and chemical burns. But not all interviewees named any specific health effect, as one interviewee expressed it: “it is a bit unclear to me which health risks there are for hexavalent chromium, I know it is not good for health.” Another simply stated “I know where to find the information [about health hazards, *authors’ note*].” An important context to consider is that, at most of the workplaces, Cr(VI) was not the exposure of highest concern, as other severe health hazards were present at the workplace and in many cases at what was perceived as more concerning. One interviewee expressed this as: “Well, I have some apprehension about other exposures than specifically chromium being high. That I do have. But not effects slap here and now, it is the long-term exposures that could be an issue. In this line of business, there are many people that have 20 to 30 years experience.”

### Interviewees’ views on the hierarchy of controls

When discussing the choice of exposure mitigation measures, our interviewees agreed that different work tasks may require different levels of mitigation. Additionally, the view on how to approach exposure reduction to Cr(VI) varied. Several interviewees expressed that there has been a general shift towards substitution as risk management measure and thus avoiding the need for later steps in the hygiene hierarchy. However, while there were examples of how Cr(VI) had been replaced to some extent at the workplaces visited, complete substitution of Cr(VI) or its source was not perceived as an option. One interviewee stated: “Our raw material is the source of hexavalent chromium, refining the raw material is the basis of our operations, hence, substitution is not possible.” Interviewees also cited goal conflicts with product quality and environmental sustainability aims. Consequences of inferior quality varied, from the risk of losing customers to safety issues for future users and shorter product life.

One interviewee stated that “Process ventilation is the alpha and omega [of exposure reduction, *authors’ note*] when substitution is not an option.” Several workplaces had recently invested in improving the general ventilation and some in the enclosure of processes. Having mobile tasks or changes in the nature and size of products were cited by others as obstacles to enclosure.

Nevertheless, when describing possible future responses in case measurements would show high levels of inhalable Cr(VI) at the workplaces, implementing more frequent use or a more protective type of RPE was in most cases the first option raised. The reliance on RPE is also illustrated by one interviewee observing a general trend of workplace risk assessments increasingly identifying the need for more layers of protection—resulting in more PPE is required today despite increased awareness of the hierarchy of controls.

### Interviewees’ expressed information needs

When asked about their information needs, or topics that would be useful to cover in future information targeting workplaces where Cr(VI) exposure occurs, not all interviewees offered suggestions. Nevertheless, five key topics emerged:

**Origin of Cr(VI) exposures**: Interviewees found it important to describe where, how, and why Cr(VI) exposures occur at workplaces. This information would provide guidance for actors not yet having mapped their own Cr(VI) sources and exposures.

**Interpretation of exposure assessments**: Interviewees requested guidance on the interpreting exposure assessments. This theme included guidance on how to interpret the overall numbers and information about factors in sampling procedure and sampling equipment influencing the validity of the data.

**General situation in Sweden**: Interviewees expressed a desire for more information on exposure levels at Swedish workplaces, including both similar and dissimilar companies to their own. Some indicated that they wished to compare their performance against other actors in the market, for instance: “I’d like to know how it may look at the other places you measure [within SafeChrom, *authors’ note*], exposure levels and so on, as a benchmark for our exposure levels.”

**Best practices for risk management:** Interviewees sought practical guidance on effectively minimizing exposures, focusing on best practices for risk management and risk mitigation measures.

**Health consequences of Cr(VI) exposure**: Interviewees found it important to provide answers to questions such as “what happens in the human body when chromium is taken up?” and “which diseases can Cr(VI) cause?”

## Discussion

Through a mixed-methods approach, we mapped the understanding of Cr(VI) risks and knowledge needs among workers with potential Cr(VI) exposures and OSH managers at these workplaces. Slightly more than half of the participants had inhalable Cr(VI) exposures above the LOD. Furthermore, RBC-Cr indicated that a similar share had a contribution from occupational exposure. Being a body-burden measure and representing a longer exposure history than urine as well as being specific for Cr(VI), RBC-Cr is the best proxy for exposure. Of our participants, 35% reported to perceive to be at no or low risk due to their Cr(VI) exposure, 47% to be at some or large risk and 18% stated to be unsure. Notably, participants’ reported risk perception did not correlate with any of the exposure measures in air, blood or urine.

A systematic review by (Verdonck et al. 2021) found support for exposure reduction measures such as technical barriers, PPE, task rotation, or shortening shifts. Saber et al. (2025) also emphasized the need to adhere to the hierarchy of controls. Our observations showed that enclosure was less frequently used than the less preferred measures of process ventilation and PPE (according to AFS – Swedish Work Environment Provisions 2023a). The interviewees perceived challenges to eliminating or substituting the source of Cr(VI) exposures and several also mentioned practical difficulties with implementing enclosure. In the context of the exposure:risk relationship for Cr(VI) and our LOD for inhalable Cr(VI), measurable air concentrations in this study correspond to exposures that in the long-term would exceed the low risk level. Therefore, use of RPE can be justified for the medium and high exposure groups, if the earlier measures in the hierarchy of controls are already in place. Participants whose inhalable Cr(VI) was above the LOD were somewhat more likely to use RPE (Table 2), but 40% did not use RPE on the sampling day, and 13% reported to never use RPE. Additionally, the occupational hygienists’ observations indicate that 2 out of 5 workers using RPE do so incorrectly, and only two of the participants reported having performed a fit-test. It can be noted that from January 2025, fit-testing of tight-fitting RPE is mandatory in Sweden (AFS 2023b).

Seventy percent of participants reported to have received workplace safety information and 64% responded that they have the knowledge to work safely. Similarly, 63% reported that the workplace provides conditions for safe work. This leaves a notable proportion reporting not being sufficiently informed or given the conditions for safe work, indicating a general need for repeated OSH training.

Regarding knowledge about potential health effects, lung cancer was identified by 92% of participants, which is similar to the 89% of surveyed bath platers identifying Cr(VI) as a lung carcinogen in Sadhra et al. (2002). Our interviewees, on the other hand, generally stated to have limited knowledge about the health hazards of Cr(VI) and were less likely to name specific health effects when responding to an open-ended interview question. There could thus be a recall effect by the questionnaire prompts. For instance, Pui et al. (2017) found that 57% of exposed workers identified diesel exhaust as a carcinogen unprompted, increasing to 77% when prompted. Yet only 40% of our participants correctly identified two or more health outcomes. Information campaigns and safety training may assist in developing fuller understandings of the health risks of Cr(VI) and safe working practices, as also suggested by Verdonck et al. (2021).

Despite reporting awareness of the lung cancer risk, participants judged their risk as low and there was no correlation between reported risk perception and their measured exposures. Our results similarly show that interviewees’ expectations of low exposures were not always met, and occupational hygienists’ assessments of the sufficiency of engineering controls were not always confirmed. As noted in previous research, both workers and OSH experts are poor judges of exposure without measurements (Vadali et al. 2012; Arnold et al. 2016; Coşkun Beyan et al. 2023).

Tangibly sensing an exposure, such as smelling it or seeing smoke/mist, may influence risk perception (Hambach et al. 2011; Persaud and Provost 2018) and/or preventive action (Meijman et al. 1996; Pui et al. 2017). Indeed, participants from bath plating companies were less likely to perceive themselves to be at some or large risk than participants from other sectors, which could potentially connect to the absence of general metal fumes as produced in smelting, casting, or welding. There are reports of a threshold for respiratory irritation from Cr(VI) at exposures in the range of single µg/m^3^ (SCOEL 2017); few of our participants are thus expected to experience a sensory response specifically to Cr(VI). Further research on how employees perceive risks with non-threshold carcinogens and how these perceptions correlate with knowledge about hazards and personal exposure levels would be interesting. In particular in relation to how organizations can implement targeted interventions to enhance workplace safety practices.

Not all interviewees expressed specific information needs during the second interview, which may illustrate that they were more concerned about other types of exposures or that they do not have a clear picture of what information would add to their knowledge. Interviewees who expressed information needs sought guidance on understanding exposures and its consequences as well as how to effectively reduce it.

Interviewees expressed a desire to know more about exposure levels at other companies as a benchmark for evaluating their own levels, indicating a potential for a peer influence on exposure reduction efforts. Possibly, a public database of exposure levels in critical sectors could function as a driving force for improvement. However, this would require companies to take the initiative in performing measurements. A study on OSH experts’ motivations for conducting workplace measurements found that motivations depended on the OSH experts’ role (Petterson-Strömbäck et al. 2006). Regardless of their role, OSH experts were prompted to measure when they expected “sufficiently high” exposures, suggesting that measurements are used to confirm suspected high exposure rather than to investigate unknown situations. Furthermore, Deubner et al. (2024) noted that exposure measurements were frequently suggested by ECHA as additional measures to applications for authorization for substances of very high concern. External demands seem to be important for motivating exposure measurements.

A limitation of our cross-sectional study is that we cannot determine if the working conditions during the measurement day, or the periods reflected by the urine-Cr and RBC-Cr are representative of participants’ full working life. Additionally, the notions of low- and high risk are subjective. Although we related the air concentrations to available policy on high and low-risk levels (ACSH 2022), we lack a quantitative benchmark for what the studied worker population considers a high risk. Factors such as delay discounting and unrealistic optimism may attenuate perceived risks. We may also have a bias towards lower risk perception due to the healthy worker effect, as it is possible that workers experiencing respiratory irritation, developing other adverse health effects, or having a tendency towards higher perception of risks from Cr(VI) exposure are less likely to start and/or remain in these occupations. Furthermore, we aimed to recruit only current non-smokers, and the perception of an occupational source for lung-cancer risk may differ between non-smokers and smokers.

Our study covers a range of different occupational settings within Sweden with exposure to Cr(VI), with respect to company size, production, and geographical location. However, it also has a small sample size and a substantial share of missing answers to the risk-related questions. Our findings may thus have limited generalizability, but we do identify a range of knowledge gaps and needs. We may have a self-selection bias towards more proactive workplaces, as companies agreeing to participate in these kinds of studies may be more inclined towards investing in OSH.

## Conclusions

Our results point towards knowledge gaps regarding the four identified dimensions of understanding risk: understanding of the health hazards, the dose–response for those hazards, the exposure levels, and how exposure can be reduced. This is concerning as a false perception of exposures as being sufficiently low may mislead the prioritization of exposure reduction measures.

Our findings regarding perceived risk could be due to limited knowledge of exposure levels (prior to our measurements). This aligns with previous research on the difficulties of correctly assessing exposures and risks without measurements. It could also indicate a lack of knowledge about the exposure:risk relationship and/or a gap between policy on high risk level and workers’ and managers’ perception of high exposures to Cr(VI). We recommend the following information efforts:

- Improving knowledge about the health hazards of Cr(VI) and the low exposures at which cancer risk exceeds the 4 in 1,000 and 4 in 100,0000 risk levels.
- Strengthening awareness of the hierarchy of controls and knowledge of the measures’ relative efficacy in exposure reduction, as well as how to use them correctly.
- Incentivising regular exposure measurements to ensure a solid basis for risk assessment and risk management.

## Supplementary material

Supplementary material is available at *Annals of Work Exposures and Health* online.

wxaf039_suppl_Supplementary_Materials

## Data Availability

The data underlying this article cannot be shared publicly due to ethical restrictions related to participant confidentiality. Data may be made available upon reasonable request to the corresponding author, subject to approval by the appropriate ethics committee and in accordance with institutional and legal data protection requirements.
