# Supplementary material for: Challenges to estimating and managing risks with hexavalent chromium exposure: a mixed-methods study of Swedish workplaces
Source: Ann Work Expo Health. 2025 Jul 4;69(7):722–35. doi: 10.1093/annweh/wxaf039 (PMC12313444; doi:10.1093/annweh/wxaf039)
Supplement: wxaf039_suppl_Supplementary_Materials [file wxaf039_suppl_supplementary_materials.pdf]

Challenges to Estimating and Managing Risks with Hexavalent Chromium Exposure: A Mixed-Methods Study of Swedish Workplaces

Linda Schenk[a]; Malin Engfeldt[b, c], Håkan Tinnerberg[d,e], Niklas Ricklund[f], Martin Tondel[g,h], Pernilla Wiebert[a,i], Maria Albin [a,i], Karin Broberg[b]

[a] Institute of Environmental Medicine, Karolinska Institutet, Stockholm, Sweden.

[b] Division of Occupational and Environmental Medicine, Department of Laboratory Medicine, Lund University, Lund, Sweden

[c] Department of Occupational and Environmental Medicine, Region Skåne, Lund, Sweden

[d] Occupational and Environmental Medicine, Sahlgrenska University Hospital, Gothenburg, Sweden

[e] Occupational and Environmental Medicine, School of Public Health and Community Medicine, Institute of Medicine, University of Gothenburg, Gothenburg, Sweden

[f] Department of Occupational and Environmental Medicine, Faculty of Medicine and Health, Örebro University, Örebro, Sweden.

[g] Department of Occupational and Environmental Medicine, Uppsala University Hospital, Uppsala, Sweden

[h]Occupational and Environmental Medicine, Department of Medical Sciences, Uppsala University, Uppsala, Sweden

[i] Centre for Occupational and Environmental Medicine, Region Stockholm, Stockholm, Sweden

Supplementary material

S1. Translation of questionnaire items on risk management and risk perception

The following items were used for the analyses presented herein. Questions on demographic background, life-style factors and work-tasks are not listed. Reply options are indicated in italics.

Have you been fit-tested for your respirator? *Yes, when? / No.*

Have you received information, instructions, or training on a safe working environment at your workplace? *Yes/ No*

Access to hygiene facilities?

- Hand-washing *Yes/No*
- Shower? *Yes/No*
- Changing rooms? *Yes/No*

Do you feel that you know enough about how to reduce your exposure to hexavalent chromium while performing your tasks? *Yes, completely / Yes, to some extent / Undecided / No, not particularly / No, not at all*

Do you believe that your workplace provides you with the conditions required to keep your exposure to hexavalent chromium sufficiently low? *Yes, completely / Yes, to some extent / Undecided / No, not particularly / No, not at all*

What health risks do you think exposure to hexavalent chromium at work can cause? You can check multiple answers. *Osteoporosis / Cancer in the lungs and airways / Cancer in other parts of the body / Cardiovascular disease / Eczema / Other lung diseases, such as asthma and COPD*

How do you assess the risk of developing a disease due to exposure to hexavalent chromium in your work? *No risk / Small risk / Some risk / Large risk / Very large risk / Unsure*

S2. Translation of semi-structured interview guide

The guide has been translated from Swedish, the translation is kept close to the original phrasing, with some additions to clarify distinctions between the singular and plural "you", where the plural refers to the workplace/company. Text in italics indicates prompts that were used if needed to guide the interviewee.

Note that results from the interviews are also presented elsewhere.

First Interview

Introduction and opening formalities

Tell me a little about yourself and your [*singular*] job.

How are occupational health and safety issues included in your [*singular*] job?

At your workplace, how are decisions made about occupational health and safety measures? *Which actors are involved?* If you [*singular*] think about the most recent measure your workplace implemented, how did it go from proposal to execution? What is your [*singular*] view on the hierarchy of controls?

What drivers do you [*singular*] see as important for your [*plural*] work with occupational safety and health? How do the regulations from the Swedish Work Environment Authority fit into this? Which regulation, if any, do you [*singular*] see as most important for your occupational health and safety work? *Why?*

What risks to workers' health do you [*singular*] see with exposure to hexavalent chromium at your workplace? *What are your [*singular*] thoughts on the cancer risk associated with hexavalent chromium?*

What is the source of hexavalent chromium at your workplace? [If applicable: Is there a safety data sheet?]

How do you [*plural*] work to reduce exposure to hexavalent chromium? What do you [*singular*] find most challenging? Which type of protective measures do you [*singular*] think make the most difference for health, i.e., reduce exposure most effectively? *Why?* Which makes the least difference? *Why?*

When do you [*singular*] think it can be said that enough has been done to reduce exposure to hexavalent chromium? What is the limit for reasonably practicable at your workplace to implement to reduce exposures?

Do you use the occupational exposure limits from the Swedish Work Environment Authority in your [*singular*] work? *How?*

What is your [*singular*] opinion on occupational exposure limits? How do you [*singular*] view them as a method for reducing health risks? Are the occupational exposure limits something you use to in your work with occupational health and safety issues? *How?* What does it mean to be below an exposure limit?

What do you [*singular*] think about the exposure limit for hexavalent chromium? *How do you* [singular] *view it from the perspective of reducing health risks? How do you [*singular*] view it from the perspective of compliance?* What factors do you [*singular*] think were considered when it was set?

Now we have talked about different ways to work with occupational health and safety and exposure to hexavalent chromium. Do you [*singular*] think there is anything I have forgotten to ask about?

Closing Formalities

Second interview

Opening Formalities

Do you [*singular*] have any thoughts or comments from the previous interview?

What do you [*singular*] think about the results you [*plural*] received from our measurements? Have you [*plural*] discussed the report with the safety committee (or equivalent)? Was there anything that surprised you [*singular*]? *Tell me more.*

Since my colleagues were at your workplace and conducted measurements, have you [*plural*] implemented any changes in your [*plural*] work methods? *Which ones?* Are you [*plural*] planning any changes?

If applicable: Can you[*singular*] tell me more about why you [*plural*] made/are planning these changes? How were these decisions influenced by the report you [*plural*] received from us?

If applicable: What considerations did you [*plural*] make regarding the trade-off between the benefits and costs of these changes?

Is there anything you [*singular*] think we should take into account as we continue to develop information materials for companies with chromium exposure? What would you [*singular*] like to learn more about? Anything you [*singular*] think similar workplaces would benefit from?

Closing Formalities

S3. Supplementary results

Table S3.1 Overview of total number of respondents in each exposure category. Low was defined as air concentrations below the LOD, or biomarkers of exposure below the P95 of the control group in Jiang et al (2024). High was defined as air concentrations above 1 μg/m^3 or biomarkers of exposure above the P85 values of the exposed workers. See also methods.

	**Number of participants:**		
	**Low** (below LOD)	**Medium**	**High**
Inhalable Cr(VI) μg/m^3	51 (51)	40	22
Urinary Cr $_{\text{density adjusted}}$ pre-shift μg/L	79 (24)	15	16
Urinary Cr $_{\text{density adjusted}}$ post-shift μg/L	53 (17)	43	17
Red blood cell Cr μg/L	51 (0)	42	17

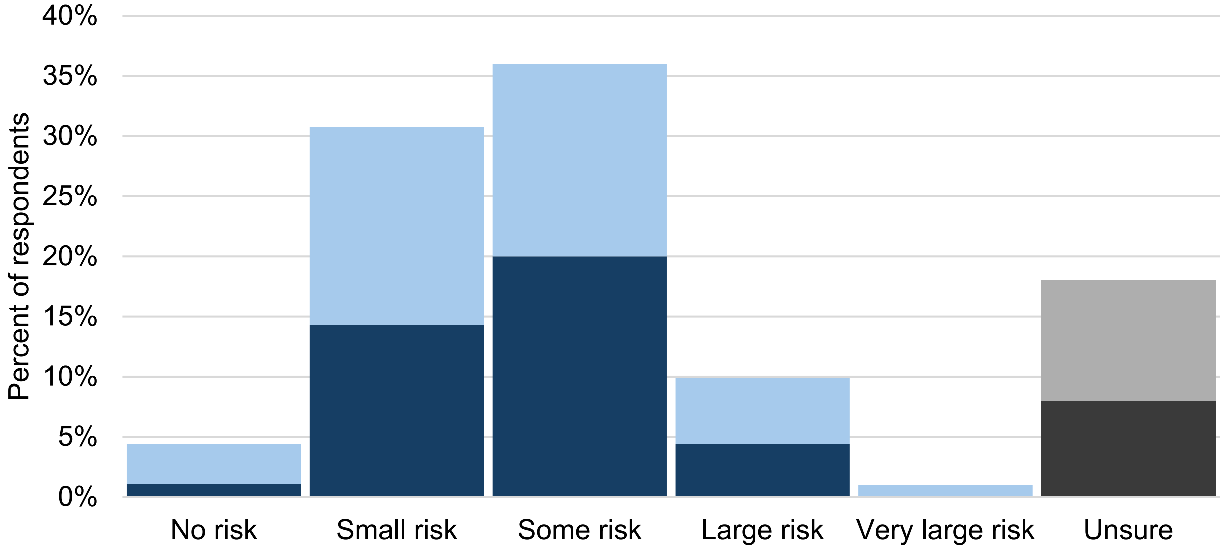

Figure S3.1 Respondents' risk judgements (n=91). The darker shade indicates respondents whose inhalable Cr(VI) levels were in the low exposure group, i.e. below the limit of detection (n=51).

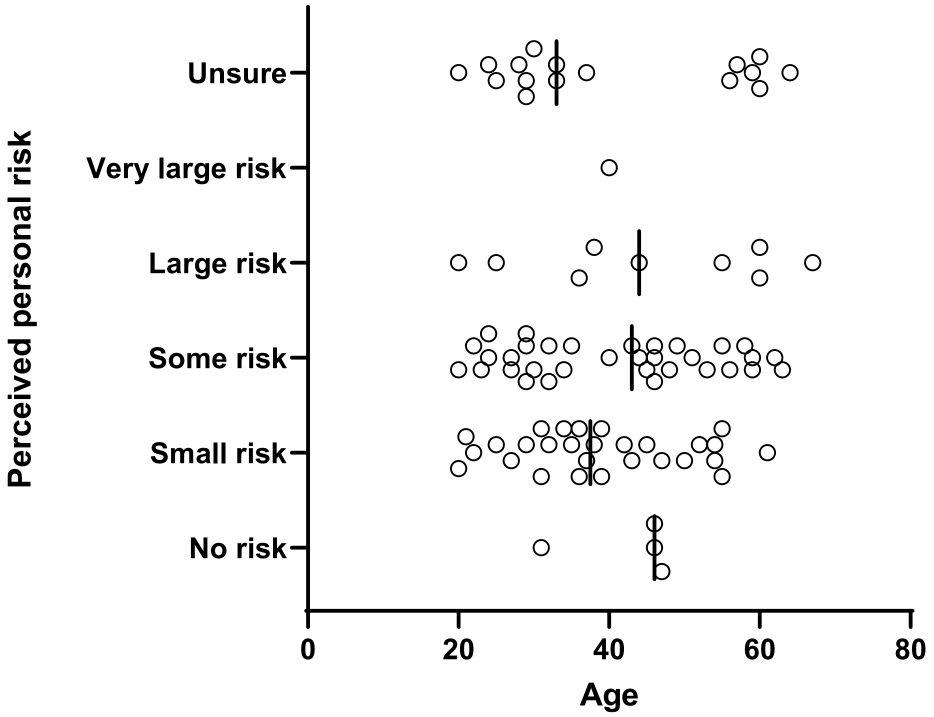

Figure S3.2 Respondents' risk judgement plotted over their age (n=91). Lines indicate median.

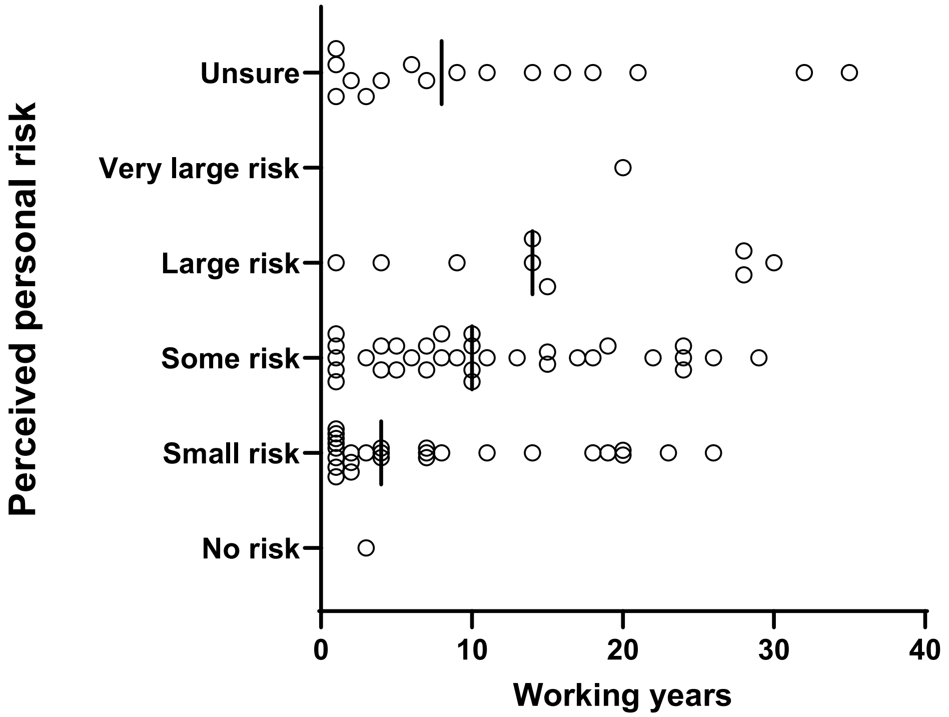

Figure S3.3 Respondents' risk judgement plotted over their years at current position (n=87). Lines indicate median. Stuart's τ_c indicates a weak positive association (τ_c=0.262, 95%CI 0.0894-0.434), excluding unsure participants.

Table S3.2 Relative risk of perceiving to be at some or high risk due to Cr(VI) exposure for different workplace factors. Modified Poisson regression with the Wald-type approximation and boot strapped confidence intervals (n=1000). All models are unadjusted, excluding participants who reported to be unsure of their personal risk.

Workplace factor	Exp. coefficient	SE	95% CI	Bootstrap 95% CI
Intercept	0.5385	0.1482	0.1482-0.4027	-
Low air exposure	1.1349	0.1991	0.7682-1.6768	0.7683-1.7143
Intercept	0.6122	0.1137	0.4900-0.7651	-
Medium air exposure	0.8167	0.2267	0.5237-1.2735	0.4921-1.2371
Intercept	0.5645	0.1115	0.4537-0.7025	-
High air exposure	1.0901	0.2460	0.6731-1.7655	0.6041-1.7196
Intercept	0.6053	0.1310	0.4682-0.7824	-
Low urine level	0.8931	0.2003	0.6031-1.3226	0.5944-1.3192
Intercept	0.5652	0.1293	0.4387-0.7283	-
Medium urine level	1.0371	0.2026	0.6972-1.5428	0.6867-1.5203
Intercept	0.5606	0.1090	0.4528-0.6941	-
High urine level	1.1892	0.2597	0.7149-1.9783	0.6141-3.3498
Intercept	0.5000	0.1543	0.3695-0.6766	-
Low blood level	1.3125	0.2004	0.8861-1.9441	0.8760-2.0029
Intercept	0.6341	0.1186	0.5026-0.8001	-
Medium blood level	0.7646	0.2151	0.5016-1.1655	0.4737-1.1591
Intercept	0.5692	0.1079	0.4607-0.7033	-
High blood level	0.9760	0.3171	0.5243-1.8169	0.4610-1.5719
Intercept	0.6667	0.0891	0.5599-0.7939	-
Bathplating (sector)	0.1250	0.9616	0.0190-0.8230	0.0190-0.5138
Intercept	0.3846	0.2481	0.2365-0.6254	-
Manufacture/processing of metal products (sector)	1.7510	0.2673	1.0370-2.9566	1.1074 -3.1111
Intercept	0.5522	0.1100	0.4451-0.6851	-
Steel production (sector)	1.3581	0.2319	0.8621-2.1395	0.7331-5.3335
Intercept	0.6176	0.0954	0.5123-0.7447	-
Machining (task)	0.2313	0.9307	0.0373-1.4335	0.0273-0.8572
Intercept	0.5957	0.1202	0.4707-0.7539	-
Process operator (task)	0.8992	0.2139	0.5923-1.3653	0.5652-1.3434
Intercept	0.4375	0.1637	0.1637-0.3174	-
Welding (task)	1.8624	0.1876	1.2894-2.6902	1.2894-2.5263
Intercept	0.5556	0.2108	0.2108-0.3675	-
Sufficient engineering controls	1.0421	0.2392	0.6521-1.6653	0.6879-1.7697
Intercept	0.5517	0.1184	0.4375-0.6958	-
Never uses RPE	1.1728	0.2147	0.7700-1.7864	0.7143-1.7579
Intercept	0.4872	0.1643	0.3531-0.6723	-
Only uses PAPR	1.3684	0.2022	0.9207-2.0339	0.9212-2.1464
Intercept	0.6604	0.0985	0.5444-0.8010	-
Uses other types of RPE	0.5506	0.2987	0.3066-0.9889	0.2920-0.9217
Intercept	0.6923	0.1307	0.5358-0.8945	-
Knowledge sufficient: yes	0.7370	0.1915	0.5063-1.0727	0.4810-1.1013
Intercept	0.5469	0.1138	0.4376-0.6835	-

Workplace factor	Exp. coefficient	SE	95% CI	Bootstrap 95% CI
Knowledge sufficient: unsure	1.3299	0.2169	0.8694-2.0343	0.7562-2.8063
Intercept	0.5500	0.1168	0.4375-0.6915	-
Knowledge sufficient: no	1.2121	0.2167	0.7926-1.8536	0.7116-1.8182
Intercept	0.7200	0.1247	0.5639-0.9194	-
Safe conditions: yes	0.6944	0.1886	0.4799-1.0049	0.4800-1.0285
Intercept	0.5873	0.1056	0.4775-0.7224	-
Safe conditions: unsure	0.8514	0.3074	0.4661-1.5551	0.4364-1.3826
Intercept	0.5000	0.1270	0.3898-0.6413	-
Safe conditions: no	1.8462	0.1501	1.3755-2.4778	1.2484-4.0880

PAPR - Powered Air Purifying Respirator; RPE – Respiratory Protective Equipment
